# Accuracy and safety of percutaneous CT-guided pancreatic biopsies

**DOI:** 10.1007/s11547-025-02087-8

**Published:** 2025-09-19

**Authors:** Thomas J. Vogl, Hamzah Adwan, Leonhard Mann, Ibrahem Al Haj Ibrahem

**Affiliations:** 1 Clinic for Radiology and Nuclear Medicine, University Hospital Frankfurt, Johann Wolfgang Goethe University, Frankfurt, Germany; 2 Department of Neuroradiology, University Hospital Frankfurt, Johann Wolfgang Goethe University, Frankfurt, Germany

**Keywords:** Interventional radiology, CT-guided pancreatic biopsy, Accuracy, Safety

## Abstract

**Purpose:**

To assess the accuracy and safety of CT-guided percutaneous core-needle biopsies of pancreatic lesions suspected to be malignant based on MRI or CT scans.

**Materials and methods:**

This retrospective study analyzed CT-guided percutaneous biopsies of suspicious pancreatic lesions performed at our university hospital. Biopsy was performed using a 17G coaxial needle and an 18G core biopsy needle. Data on patient characteristics, lesions, procedures, and histologic results were recorded.

**Results:**

A total of 90 patients (58.9% males, mean age 65 ± 12.2 years) underwent CT-guided percutaneous biopsies. The lesions had a mean size of 36.8 ± 12.2 mm and were predominantly located in the pancreatic head 61.1% (55/90). Technical success was achieved in all biopsies 100% (90/90). Most procedures 96.7% (87/90) were performed using direct access routes, while 3.3% (3/90) required indirect transhepatic or transgastric approaches. Among the biopsies, 65.6% (59/90) confirmed malignancy, with adenocarcinoma as the most common malignant subtype representing 55.6% (50/90) of all cases. The rate of non-malignant findings was 26.7% (24/90) including chronic pancreatitis at a rate of 5.6% (5/90) as well as pancreatic pseudocysts and pancreatic cystadenoma each at a rate of 2.2% (2/90), among others. A total of seven cases were identified as false negatives confirmed, but malignancy was later confirmed after re-biopsy or surgery. The initial diagnostic accuracy was 92.2% (83/90). The rate of major complications was 1.1% (1/90), and a total of two minor complications at a rate of 2.2% were observed.

**Conclusion:**

This study shows that CT-guided pancreatic biopsy is a safe procedure with high diagnostic accuracy.

## Introduction

In 2022, pancreatic cancer is the sixth leading cause of cancer-related deaths for both women and men and the twelfth most commonly diagnosed cancer worldwide, with higher rates in countries with higher Human Development Index compared to countries with lower Human Development Index [1]. There are several risk factors for pancreatic cancers including smoking, diabetes mellitus, excessive alcohol consumption, pancreatitis, among others [2]. Adenocarcinoma, the most common form of pancreatic cancer, is highly aggressive because of the late diagnosis and restricted therapy response [3]. The high mortality and incidence of pancreatic cancer highlight the importance of thoroughly evaluating pancreatic masses to confirm or rule out malignancy, ensuring timely and appropriate clinical management. For the diagnosis of pancreatic cancer, various imaging modalities can be applied, including computed tomography (CT), magnetic resonance imaging (MRI), and endoscopic ultrasound (EUS) [4]. However, in some cases a biopsy should be performed for various reasons such as distinguishing malignant from non-malignant pancreatic masses [5], and to determine the histopathological grading of malignant tumors, which has therapeutic consequences [6]. Multiple biopsy techniques are available to obtain tissue samples for histopathological evaluation, including percutaneous, endoscopic, and surgical approaches [5]. Surgical biopsy is more invasive and riskier compared to the cost-effective image-guided biopsy such as endoscopic ultrasound-guided fine-needle aspiration (EUS-FNA) and percutaneous CT-guided biopsy [7].

This study focuses on assessing the safety and outcome of CT-guided percutaneous core-needle biopsies of indeterminate pancreatic masses.

## Materials and methods

This retrospective study was approved by the institutional review board of our university hospital and conducted according to the ethics guidelines of the Declaration of Helsinki. An informed consent to study participation was waived, due to the retrospective design.

We conducted a retrospective review of all patients referred to our clinic of radiology for pancreatic biopsies until December 2023. All patients had undergone prior cross-sectional imaging (MRI or CT) that suggested the presence of potentially malignant pancreatic lesions.

### Inclusion criteria

Patients with imaging findings indicative of potentially malignant pancreatic lesions were eligible for biopsy. These findings included masses or irregular borders, among others, identified on MRI or CT.

### Exclusion criteria

- Patients with significant comorbidities, such as bleeding disorders or severe cardiac and respiratory conditions, that increased the procedural risk.
- Cases with inconclusive or nonspecific imaging findings where malignancy was not strongly suspected.
- Patients who declined to provide informed consent or were medically unstable for the procedure.
- Patients under the age of 18.

### Definitions

Complications were defined based on the Society of Interventional Radiology (SIR) Classification System and divided into minor complications and major complications [8]. Technical success was reached if the biopsy was performed and completed as planned with tissue sampling for histopathological diagnosis [9, 10].

### Biopsy procedure

**Fig. 1:**
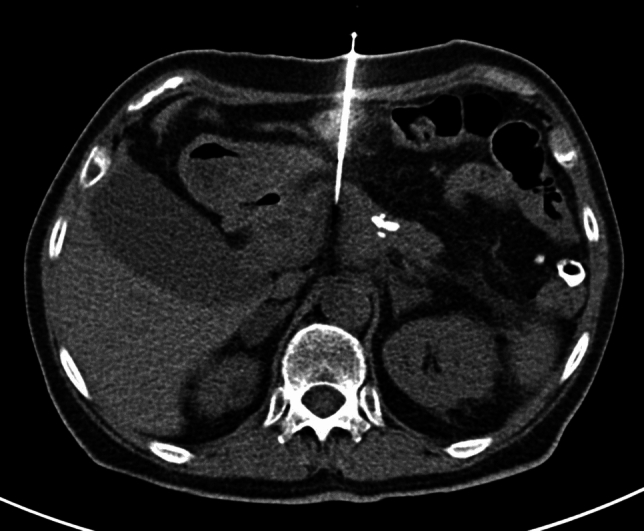
Percutaneous CT-guided biopsy of a pancreatic lesion using a direct anterior access. A non-enhanced axial CT image shows the needle inserted through the anterior abdominal wall, directed toward the lesion in the body of the pancreas

**Fig. 2:**
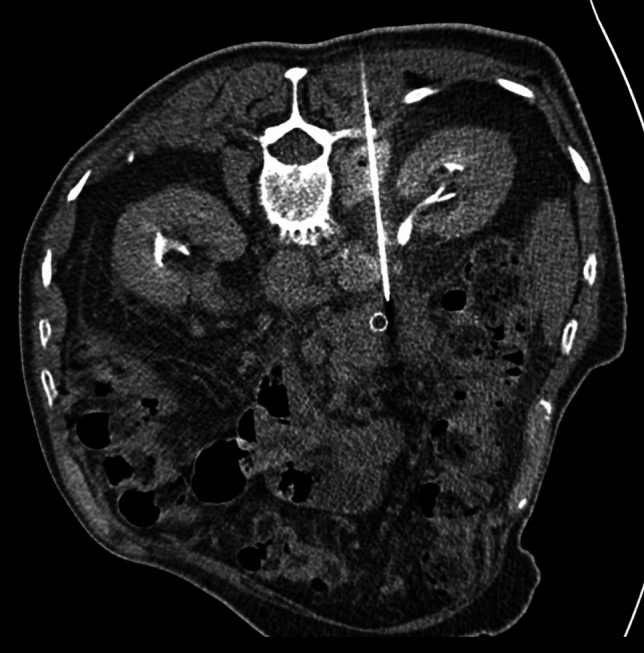
The axial CT image shows the biopsy needle inserted posteriorly toward a pancreatic head lesion. The anterior approach was avoided due to interposed bowel loops. Additionally, contrast agent was administered to visualize the ureter to avoid its injury by the needle. The histopathological diagnosis was moderately differentiated adenocarcinoma

**Fig. 3:**
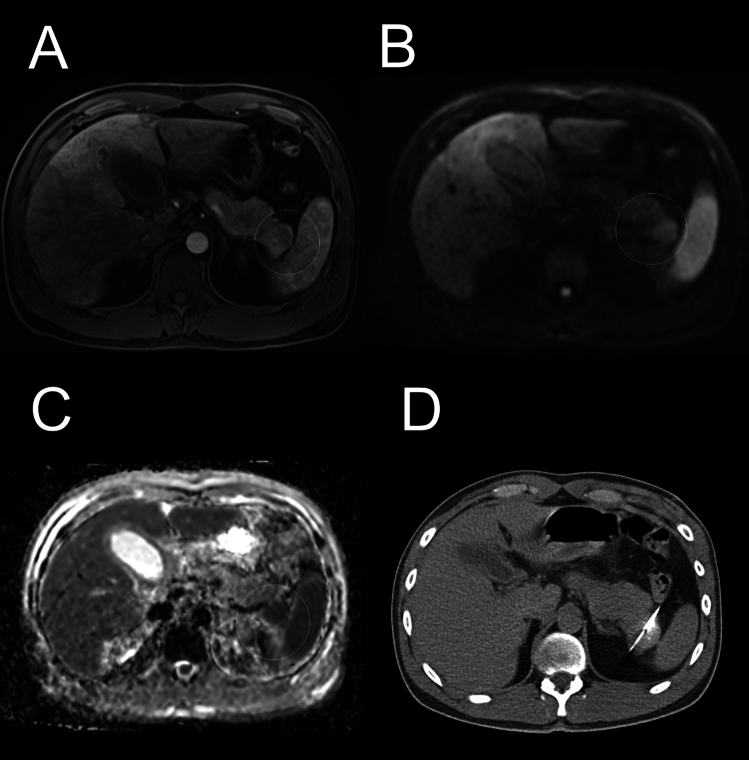
Contrast-enhanced T1-weighted image in the arterial phase shows a mass lesion in the pancreatic tail (Image **A**). The mass showed a hyperintense signal on diffusion-weighted imaging (Image **B**) with corresponding hypointense signal on the apparent diffusion coefficient map (Image **C**) confirming diffusion restriction, further supporting the lesion’s malignant nature. Image **D**: CT-guided biopsy using lateral approach targeting the lesion in the pancreatic tail. Histological analysis confirmed the diagnosis of a neuroendocrine tumor

**Fig. 4:**
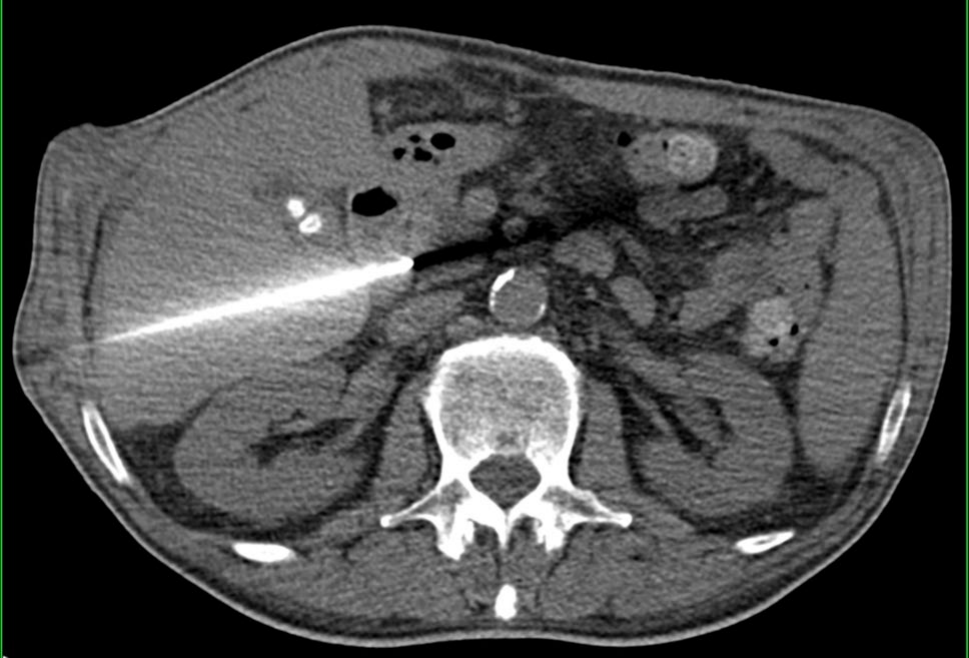
CT-guided transhepatic biopsy of a lesion in the pancreatic head. The needle trajectory passes through the liver and duodenum toward the target lesion

Before performing the biopsy, informed consent was obtained from each patient. Each case was assessed to determine the optimal biopsy access route, with direct access prioritized. A 128- or 256-slice CT scanner (Siemens, Germany) was utilized for the procedure. CT-guided biopsies were performed under local anesthesia in coaxial technique using a 17G coaxial needle and an 18G core biopsy needle (Tru-Core™). In some cases, intravenous contrast agent was administered to visualize critical structures to avoid them. Access routes included anterior (Fig. 1), posterior (Fig. 2), left lateral (Fig. 3), and right lateral approaches. Access type included direct and indirect approaches (transgastric or transhepatic), which were employed when direct access was not feasible. Figure 4 demonstrates a case with an indirect access.

### Data Collection

Patient data were collected using a standardized form to ensure consistency and comprehensive analysis across all cases. The form documented demographics (age, sex, presence of primary neoplasia), lesion characteristics (location within the pancreas, size), procedural details (access route, complications), and histopathologic findings. Lesions were classified as benign or malignant, with specific tumor types recorded for malignant lesions, including subtypes such as adenocarcinoma, neuroendocrine tumors, or other malignancies. This structured approach facilitated a thorough evaluation of the biopsy procedure and its outcomes.

### Statistical analysis

All data were analyzed using IBM SPSS Statistics software (version 30.0.0.0; IBM Corp., Armonk, NY, USA). Descriptive statistics, including frequencies and percentages, were used to summarize categorical variables. Continuous variables were described using means and standard deviations.

## Results

A total of 90 patients underwent CT-guided percutaneous biopsies, with a mean age of 65 ± 12.2 years. Of these, 58.9% (53/90) were male, and 13.3% (12/90) had previous primary tumors, most commonly lung and breast cancers. Patient demographic and clinical characteristics are summarized in Table 1. The majority, 61.1% (55/90) of lesions were located in the pancreatic head (Table 2), with a mean size of 36.8 ± 12.2 mm. Most procedures, 96.7% (87/90), were performed using direct access, with 3.3% (3/90) requiring indirect transhepatic or transgastric approaches. The most biopsies were performed via the anterior access route at a rate of 76.7% (69/90) followed by left lateral approach at a rate of 16.7% (15/90).

**Table 1:** Baseline demographic and clinical characteristics of the whole patient cohort (n = 90)

Variable	Value
Mean age	65 ± 12.2 years
Male sex, n (%)	53 (58.9)
History of prior malignancy, n (%)	12 (13.3)
Mean lesion size (SD)	36.8 ± 12.2 mm
Access route	
Anterior, n (%)	69 (76.7)
Left lateral, n (%)	15 (16.7)
Posterior, n (%)	4 (4.4)
Right lateral, n (%)	2 (2.2)
Access type	
Direct, n (%)	87 (96.7)
Indirect, n (%)	3 (3.3)

**Table 2:** Anatomical distribution of pancreatic lesions

Location	Frequency	Percent (of total n = 90)
Head	55	61.1%
Body	23	25.6%
Tail	12	13.3%
Total	90	100.0%

Histological findings after initial biopsy showed the majority were categorized as malignant, with 65.6% of the cases (59/90) being confirmed as malignant and 26.7% (24/90) diagnosed as non-malignant.

Among the 59 malignant cases (Table 3), the most prevalent malignant histological subtype was adenocarcinoma, identified in 55.6% (50/90) of all cases. Neuroendocrine tumor was present in 4.4% (4/90) of all cases. Both non-small cell carcinoma and solid pseudopapillary epithelial neoplasm were each identified in 2.2% of the cases, with 2 patients in each group. Melanoma was observed in 1.1% of all cases, affecting 1 patient. The mean size of malignant lesions was 36.0 ± 13.3 mm.

**Table 3:** Distribution of malignant histological subtypes

Subtype	Frequency	Percent (of total n = 90)
Adenocarcinoma	50	55.6%
Neuroendocrine tumor	4	4.4%
Non-small cell carcinoma	2	2.2%
Solid pseudopapillary epithelial neoplasm	2	2.2%
Melanoma	1	1.1%

Chronic pancreatitis was diagnosed in 5.6% of the total cases (5/90). Both pancreatic cystadenoma and pancreatic pseudocysts were each found in 2.2% of the total cases as shown in Table 4. The mean size of non-malignant lesions was 30.0 ± 20.5 mm.

**Table 4:** Distribution of non-malignant histological findings

Finding	Frequency	Percent (of total n = 90)
Chronic pancreatitis	5	5.6%
Pancreatic cystadenoma	2	2.2%
Pancreatic pseudocyst	2	2.2%
Other	15	16.7%

A total of seven patients with a mean lesion size of 30.0 ± 7.7 mm were classified as false negatives, where the initial biopsy was negative, but malignancy was later confirmed, after subsequent biopsies or surgical resection via pancreaticoduodenectomy. The initial diagnostic accuracy was 92.2% (83/90).

The overall complication rate was 3.3% (3/90). One patient at a rate of 1.1% (1/90) had severe bleeding, which was treated by embolization. Two patients at a rate of 2.2% (2/90) had mild bleeding. None of the patients had infection or abscesses.

## Discussion

This retrospective study highlights the significant role of CT-guided percutaneous core-needle biopsy in the diagnosis of pancreatic lesions suspected to be malignant. The most cases were malignant at a rate of 65.6%, among the ninety patients evaluated, with adenocarcinoma accounting for the most malignant cases. Technical success was achieved in all cases, and the rate of initial diagnostic accuracy was 92.2% (83/90). We observed only one major complication at a rate of 1.1% (1/90).

Hwang et al. included in their study a total of 139 patients with pancreatic and peripancreatic solid masses, who underwent EUS-FNA [11]. The mean size of the lesions in their study was 40.5 mm. They reported an overall accuracy rate of 82.7%. Notably, they also found that lesion size marginally influences EUS-FNA accuracy on multivariate analysis. In comparison, our study yielded a higher diagnostic accuracy of 92.2%, despite a slightly smaller mean size of all lesions in our cohort at 36.8 mm.

In cases of small pancreatic lesions (≤ 10 mm), Sagami et al. [12] reported a diagnostic accuracy of 91.3% for EUS-FNA. However, the technical success rate was relatively low at 80.8%. This may be primarily due to challenges regarding anatomical accessibility or inadequate visibility of the tumor.

Among the cases evaluated in our study, seven cases of biopsies were classified as false negatives, where malignancy was identified only after subsequent biopsies or surgical resection. These findings underscore the necessity of follow-up imaging and additional biopsies or even resection, when pancreatic lesions are still suspicious to be clinically and/or radiologically malignant.

CT-guided biopsy techniques have consistently demonstrated high diagnostic success in the literature. Lin et al. [13] reported a diagnostic success rate of 97.4% using a fat-transversing coaxial technique, with 85.1% of cases confirmed as malignant and a complication rate of just 4.1%. This approach minimized the injury risk by avoiding organ penetration through strategic access via surrounding fat. Similarly, Tyng et al. [14] achieved a diagnostic success rate of 98.1%, with adenocarcinoma comprising most of the malignant cases. Notably, 22.2% of procedures in their cohort employed specialized techniques such as hydrodissection and/or pneumodissection. They also applied indirect accesses in 4.8% of the cases. They only reported minor complications at a rate of 8.7%.

In our study, the high prevalence of malignant findings, especially adenocarcinoma in our cohort, aligns with these findings, reaffirming the reliability of CT-guided biopsy in malignancy diagnosis. In terms of safety, CT-guided biopsy proved to be a highly effective and minimally invasive method for obtaining tissue samples, with no major complications reported in our study, despite the potential risks associated with indirect access routes, such as transgastric or transhepatic pathways.

Specialized access routes have also been explored for difficult cases. Sofocleous et al. [15] included a total of 55 patients, who underwent 58 biopsies of pancreatic or peripancreatic masses and demonstrated an 86% diagnostic accuracy using a posterior approach traversing the inferior vena cava or renal vein, with no relevant complications. Although such approaches were not employed in our study, they offer valuable alternatives when conventional techniques are unfeasible.

The retrospective design and relatively small sample size of our study limit the generalizability of our findings. Additionally, the absence of direct comparisons with other biopsy techniques is a notable limitation. Future prospective studies directly comparing CT-guided with EUS-FNA, for instance, in various clinical scenarios are essential to refine diagnostic strategies and improve patient outcomes.

In conclusion, our study showed that CT-guided biopsy of suspicious pancreatic lesions is a safe, reliable, and effective diagnostic tool with high accuracy, even under the application of indirect accesses.

## References

[1] Bray F, Laversanne M, Sung H, Ferlay J, Siegel RL, Soerjomataram I, Jemal A (2024) Global cancer statistics 2022: GLOBOCAN estimates of incidence and mortality worldwide for 36 cancers in 185 countries. CA Cancer J Clin 74:229–263. 10.3322/caac.21834 38572751 10.3322/caac.21834

[2] Klein AP (2021) Pancreatic cancer epidemiology: understanding the role of lifestyle and inherited risk factors. Nat Rev Gastroenterol Hepatol 18:493–502. 10.1038/s41575-021-00457-x 34002083 10.1038/s41575-021-00457-x PMC9265847

[3] Sarantis P, Koustas E, Papadimitropoulou A, Papavassiliou AG, Karamouzis MV (2020) Pancreatic ductal adenocarcinoma: Treatment hurdles, tumor microenvironment and immunotherapy. World J Gastrointest Oncol 12(2):173–181. 10.4251/wjgo.v12.i2.173 32104548 10.4251/wjgo.v12.i2.173 PMC7031151

[4] Puckett Y, Garfield K (2025) Pancreatic Cancer. [Updated 2024 Sep 10]. In: StatPearls [Internet]. Treasure Island (FL): StatPearls Publishing; Available from: https://www.ncbi.nlm.nih.gov/books/NBK518996/30085538

[5] Mittal A, Le A, Kahlam A, Haider SF, Prasath V, Khrais A, Chokshi R (2023) Pancreatic cancer biopsy modalities: comparing insurance status, length of stay, and hospital complications based on percutaneous, endoscopic, and surgical biopsy methods. Cureus 15:e39660. 10.7759/cureus.39660 37388621 10.7759/cureus.39660 PMC10306347

[6] Gwoździewicz K, Studniarek M, Czarnowska-Cubała M, Pieńkowska J, Markiet K (2023) Usefulness of core biopsy in diagnosis of pancreatic tumours. Pol J Radiol 88:e529–e534. 10.5114/pjr.2023.132890 38125812 10.5114/pjr.2023.132890 PMC10731441

[7] Bao W, Zeng K, Huang J, Yu B, Gou Z, Lu Q (2025) Comparative analysis of different biopsy techniques for pancreatic lesions in diagnostic value, safety, and cost-effectiveness. Quant Imaging Med Surg 15(5):4375–4386. 10.21037/qims-2024-2670 40384725 10.21037/qims-2024-2670 PMC12084684

[8] Sacks D, McClenny TE, Cardella JF, Lewis CA (2003) Society of Interventional Radiology clinical practice guidelines. J Vasc Interv Radiol 14(Suppl 2):S199–S202. 10.1097/01.rvi.0000094584.83406.3e 14514818 10.1097/01.rvi.0000094584.83406.3e

[9] Aslan HS, Alver KH (2025) US-Guided percutaneous core needle biopsy via the complete transhepatic approach: a reliable option for deep abdominal lesions. Abdom Radiol (NY). 10.1007/s00261-025-04958-0 40285794 10.1007/s00261-025-04958-0 PMC12568807

[10] Zhang Y, Huang H, Cao S et al (2021) Clinical value of an electromagnetic navigation system for CT-guided percutaneous lung biopsy of peripheral lung lesions. J Thorac Dis 13(8):4885–4893. 10.21037/jtd-21-395 34527327 10.21037/jtd-21-395 PMC8411150

[11] Hwang CY, Lee SS, Song TJ, Moon SH, Lee D, Park DH, Seo DW, Lee SK, Kim MH (2009) Endoscopic ultrasound guided fine needle aspiration biopsy in diagnosis of pancreatic and peripancreatic lesions: a single center experience in Korea. Gut Liver 3(2):116–121. 10.5009/gnl.2009.3.2.116 20431733 10.5009/gnl.2009.3.2.116 PMC2852702

[12] Sagami R, Nakahodo J, Minami R, Yamao K, Yoshida A, Nishikiori H, Takenaka M, Mizukami K, Murakami K (2024) True diagnostic ability of EUS-guided fine-needle aspiration/biopsy sampling for small pancreatic lesions ≤10 mm and salvage diagnosis by pancreatic juice cytology: a multicenter study. Gastrointest Endosc 99(1):73–80. 10.1016/j.gie.2023.08.006 37598865 10.1016/j.gie.2023.08.006

[13] Lin CY, Ou MC, Liu YS, Chuang MT, Shan YS, Tsai HM, Wang CK, Tsai YS (2017) A CT-guided fat transversing coaxial biopsy technique for pancreatic lesion biopsy that avoids major organs and vessels. Saudi J Gastroenterol 23(6):341–347. 10.4103/sjg.SJG_199_17 29205187 10.4103/sjg.SJG_199_17 PMC5738796

[14] Tyng CJ, Almeida MF, Barbosa PN, Bitencourt AG, Berg JA, Maciel MS, Coimbra FJ, Schiavon LH, Begnami MD, Guimarães MD, Zurstrassen CE, Chojniak R (2015) Computed tomography-guided percutaneous core needle biopsy in pancreatic tumor diagnosis. World J Gastroenterol 21(12):3579–3586. 10.3748/wjg.v21.i12.3579 25834323 10.3748/wjg.v21.i12.3579 PMC4375580

[15] Sofocleous CT, Schubert J, Brown KT, Brody LA, Covey AM, Getrajdman GI (2004) CT-guided transvenous or transcaval needle biopsy of pancreatic and peripancreatic lesions. J Vasc Interv Radiol 15(10):1099–1104. 10.1097/01.RVI.0000130815.79121.EC 15466796 10.1097/01.RVI.0000130815.79121.EC

